# Changes in Near-Infrared Spectroscopy After Congenital Cyanotic Heart Surgery

**DOI:** 10.3389/fped.2018.00097

**Published:** 2018-04-13

**Authors:** Judith Ju-Ming Wong, Ching Kit Chen, Rajesh Babu Moorakonda, Olivia Wijeweera, Tracy Yi Shuen Tan, Masakazu Nakao, John Carson Allen, Tsee Foong Loh, Jan Hau Lee

**Affiliations:** ^1^Children’s Intensive Care Unit, Department of Paediatric Subspecialties, KK Women’s and Children’s Hospital, Singapore, Singapore; ^2^Duke-NUS Medical School, Singapore, Singapore; ^3^Cardiology Services, Department of Paediatric Subspecialties, KK Women’s and Children’s Hospital, Singapore, Singapore; ^4^Singapore Clinical Research Institute, Singapore, Singapore; ^5^Duke-NUS Medical School, Centre for Quantitative Medicine, Singapore, Singapore; ^6^Department of Paediatric Anaesthesia, KK Women’s and Children’s Hospital, Singapore, Singapore; ^7^Department of Paediatric Cardiothoracic Surgery, KK Women’s and Children’s Hospital, Singapore, Singapore

**Keywords:** congenital heart disease, congenital heart surgery, children, near-infrared spectroscopy, perioperative care, pediatric, regional oximetry

## Abstract

**Background:**

Since oxygen saturation from pulse oximetry (SpO_2_) and partial pressure of arterial oxygen (PaO_2_) are observed to improve immediately after surgical correction of cyanotic congenital heart disease (CHD), we postulate that cerebral (CrO_2_) and somatic (SrO_2_) oximetry also improves immediately post-correction. We aim to prospectively examine CrO_2_ and SrO_2_, before, during, and after surgical correction as well as on hospital discharge in children with cyanotic CHD to determine if and when these variables increase.

**Methods:**

This is a prospective observational trial. Eligibility criteria included children below 18 years of age with cyanotic CHD who required any cardiac surgical procedure. CrO_2_ and SrO_2_ measurements were summarized at six time-points for comparison: (1) pre-cardiopulmonary bypass (CPB); (2) during CPB; (3) post-CPB; (4) Day 1 in the pediatric intensive care unit (PICU); (5) Day 2 PICU; and (6) discharge. Categorical and continuous variables are presented as counts (percentages) and median (interquartile range), respectively.

**Results:**

Twenty-one patients were analyzed. 15 (71.4%) and 6 (28.6%) patients underwent corrective and palliative surgeries, respectively. In the corrective surgery group, SpO_2_ increased immediately post-CPB compared to pre-CPB [99 (98, 100) vs. 86% (79, 90); *p* < 0.001] and remained in the normal range through to hospital discharge. Post-CPB CrO_2_ did not change from pre-CPB [72.8 (58.8, 79.0) vs. 72.1% (63.0, 78.3); *p* = 0.761] and even decreased on hospital discharge [60.5 (53.6, 62.9) vs. 72.1% (63.0, 78.3); *p* = 0.005]. Post-CPB SrO_2_ increased compared to pre-CPB [87.3 (77.2, 89.5) vs. 72.7% (65.6, 77.3); *p* = 0.001] but progressively decreased during PICU stay to a value lower than baseline at hospital discharge [66.9 (57.3, 76.9) vs. 72.7% (65.6, 77.3); *p* = 0.048].

**Conclusion:**

CrO_2_ and SrO_2_ did not increase after corrective surgery of cyanotic CHD even up to hospital discharge. Future larger studies are required to validate these findings. (This study is registered with ClinicalTrials.gov ID: NCT02417259.)

## Introduction

Adoption of near-infrared spectroscopy (NIRS) is increasing in pediatric cardiac anesthesia and pediatric intensive care units (PICUs) across North America and Europe (1). The two most common sites for regional NIRS are cerebral (CrO_2_) and somatic (SrO_2_) oximetry. Proposed utility of NIRS include: (1) pre-operative risk stratification; (2) monitoring for uncommon events; and (3) guide physiologic interventions (2). However, significant debate remains on the interpretation of NIRS monitoring because of the lack of universally accepted normal/abnormal values, definition of a threshold event and standardized management algorithm (1, 3).

Despite current limitations, cerebral oximetry has been validated with other regional oxygenation measures (e.g., jugular venous bulb saturations, superior vena cava saturations) and global oxygenation measures (e.g., serum lactate, mixed venous saturations) (4–6). Associations between low NIRS and poor long-term neurological outcomes (e.g., neurodevelopmental scales, magnetic resonance imaging changes) have been reported (7, 8). Conversely, however, there are also studies reporting failure of NIRS to correlate with oxygenation measures or clinical outcomes (9). Moreover, the natural history of tissue oximetry over a longitudinal perioperative period in children with congenital heart disease (CHD), especially those with cyanotic CHD undergoing cardiac surgery is not well studied. Early studies reported conflicting data as to whether baseline CrO_2_ was decreased in patients with cyanotic and acyanotic CHD (10, 11). There is sparse longitudinal data across the perioperative period (12).

Since oxygen saturations from pulse oximetry (SpO_2_) and partial pressure of arterial oxygen (PaO_2_) are observed to improve immediately after surgical correction of cyanotic CHD, we postulate that CrO_2_ and somatic (SrO_2_) oximetry would also improve immediately post-correction. Therefore, we undertook this pilot study to examine CrO_2_ and SrO_2_, before, during and after surgical correction as well as on hospital discharge in these children to determine if and when these variables increase.

## Materials and Methods

### Patients

This prospective observational study was approved by the Centralized Institutional Review Board of our hospital. Both parental informed and written consent was obtained and patients were enrolled between July 2015 and June 2016 at KK Women’s and Children’s Hospital, Singapore. Eligibility criteria included patients below 18 years of age with cyanotic CHD who required any cardiac surgical procedure. These surgeries can be either full corrective or staged procedures. Exclusion criteria included premature infants of less than a corrected age of 35 weeks and congenital lactic acidosis syndromes.

### Anesthesia and Perfusion Methods

The anesthesia induction technique was at the discretion of the attending anesthetist. Inhaled anesthetic used was sevoflurane. Intravenous agents included morphine, fentanyl, ketamine, midazolam, propofol, rocuronium, and dexmedetomidine. After induction of anesthesia, standard monitoring included: central venous catheter in the right or left internal jugular vein for central venous pressure monitoring; radial or femoral artery catheter for measurement of systemic arterial blood pressure; indwelling urinary catheter for monitoring urinary output and; esophageal/nasopharyngeal and rectal temperature probes to measure core body temperature. Core cooling was achieved through the cardiopulmonary bypass (CPB) circuit. The bypass circuit was primed with packed cells. Pump flow rates were maintained at 100–150 ml/kg/min to achieve mean arterial pressure of 30–55 mmHg. Deep hypothermic circulatory arrest (DHCA) to 18°C was used at the discretion of the surgeon. Modified ultrafiltration was performed in infants and patients below 10 kg after CPB.

### Regional Oxygen Saturation Monitoring

The use of CrO_2_ and SrO_2_ in patients undergoing cardiac surgery is not routine at our center. During the study, CrO_2_ and SrO_2_ were measured in the context of a prospective observational study and not for clinical purposes.

Regional oxygen saturation was measured using the Medtronic INVOS 5100C, Boulder, CO, USA. Two site recording from CrO_2_ and SrO_2_ regions were performed before induction of anesthesia. The cerebral sensor was placed over the left or right forehead midway between the eyebrow and hairline and the somatic sensor was placed over the left or right flank at T12–L2 vertebral level. After a short period to allow for adjustments to obtain good contact, the baseline tissue oxygenation was recorded. In children who were fretful and uncooperative, the probes were placed and baseline recorded soon after induction of anesthesia. Subsequently, data were collected and downloaded to a storage device every 6 s throughout the surgery and for 48 h from admission to the PICU. A pre-discharge recording was made just prior to hospital discharge—this was done over a period of 4–6 h while the patient was asleep. If the hospital discharge was delayed the pre-discharge recording was taken whenever the attending cardiologist deemed the patient medically fit for discharge.

### Data Extraction

Intraoperative clinical data were extracted from important time-points such as time of induction, median sternotomy, start and end of CPB, aortic clamp and unclamp, and end of surgery. Clinical and laboratory variables (e.g., heart rate, arterial blood pressure, central venous pressure, temperature, urine output, arterial blood gases, and serum lactate) were measured and recorded as per routine practice in the operating theater and PICU. Acute kidney injury was defined according to the RIFLE criteria. Including those who were at Risk of kidney injury (≥1.5× baseline creatinine or urine output <0.5 ml/kg/h) (13).

### Statistical Analysis

Cerebral oximetry and SrO_2_ data were captured electronically every 6 s. These were summarized into medians [interquartile ranges (IQR)] for six time-points for comparison: (1) pre-CPB (from time of application of NIRS sensors before induction of anesthesia to time of CPB initiation); (2) on CPB (from time of initiation to end CPB); (3) post-CPB (from time of end CPB to transfer out from operating theater); (4) Day 1 PICU (from time of PICU admission for 24 h); (5) Day 2 PICU (from 24 to 48 h of PICU admission); and (6) discharge (4–6 h overnight prior to day of anticipated discharge). Categorical data were presented as counts and percentages. Continuous data were presented as medians and IQR. Differences between categorical variables were analyzed using the Fisher’s exact test. Differences between continuous variables were analyzed using the Wilcoxon’s signed rank test. Statistical analysis was performed using SAS 9.4 for Windows (SAS Institute Cary, NC, USA). All statistical tests were two-tailed, and the significance level was taken as *p* < 0.05.

## Results

**Figure 1:**
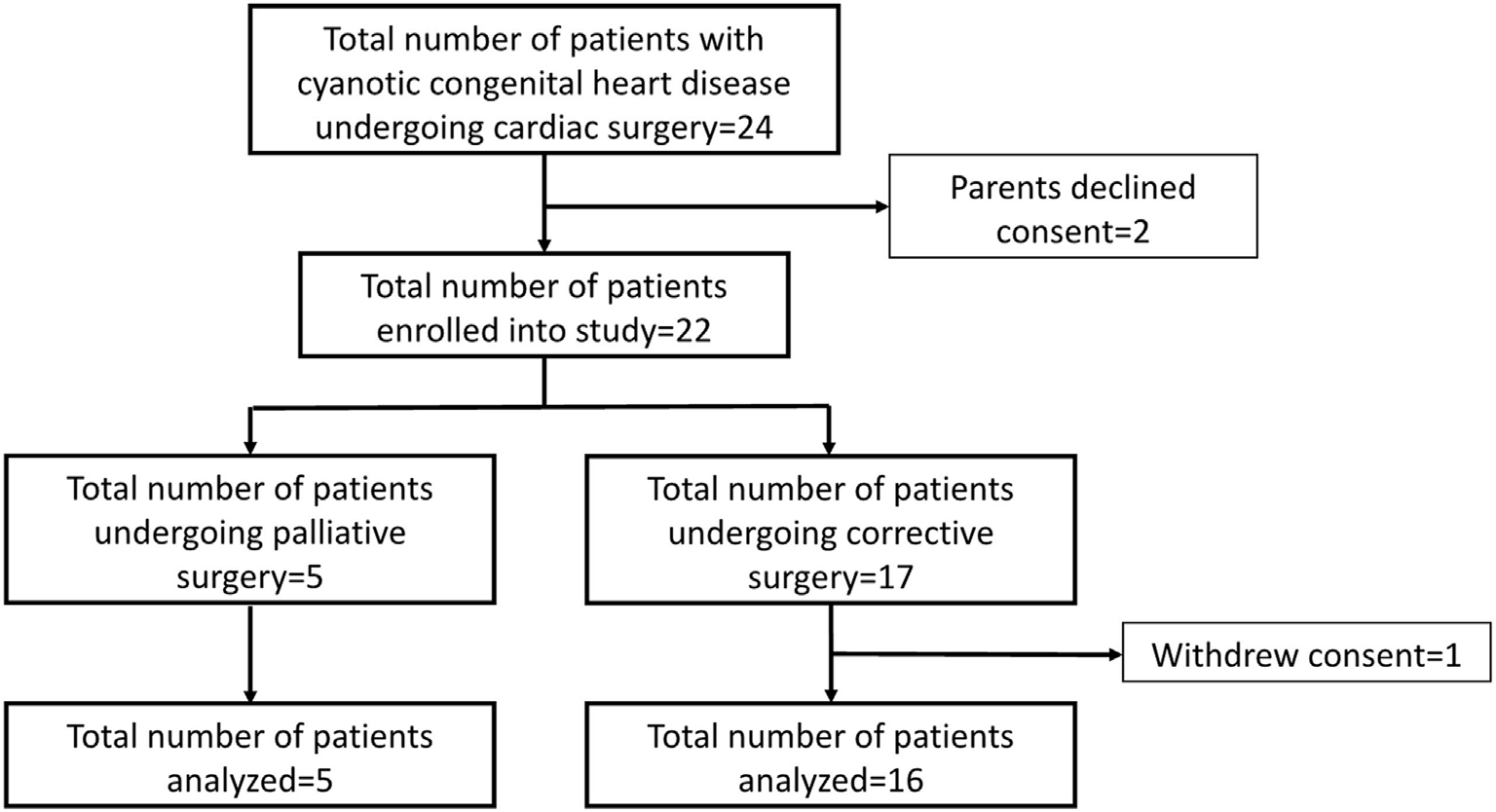
CONSORT diagram.

A total of 24 children with cyanotic CHD underwent surgery over the 1-year study period and all were approached for consent. Parents of two children declined to participate in the study and parents of another child withdrew consent after enrollment (Figure 1). The overall median age was 18.1 (7.8, 51.4) months (Table 1). Fifteen [15/21 (71.4%)] children underwent corrective surgeries. The most common surgery was repair of tetralogy of Fallot [7/21 (33.3%)] followed by the Fontan procedure [3/21 (14.3%)] (Table 2). The median CPB and aortic cross-clamp times were 187.5 (113.0, 263.5) and 80.0 (37.0, 133.0) minutes, respectively (Table 2). Only one patient (Norwood procedure) required total circulatory arrest (duration 77 min). Intraoperatively, adrenaline [20/21 (95.2%)] and milrinone [18/21 (85.7%)] were the most frequently used inotropes.

**Table 1:** Summary of patient characteristics (*N* = 21).

Patient characteristics	Corrective surgery (*N* = 15)	Non-corrective surgery (*N* = 6)	All patients (*N* = 21)
Age, months	18.7 (10.3, 51.4)	8.0 (0.4, 46.0)	18.1 (7.8, 51.4)
Weight, kg	10.5 (7.0, 14.1)	6.0 (3.0, 11.4)	8.5 (5.4, 13.5)
Male gender	6 (40.0)	3 (50.0)	9 (42.9)

**Ethnicity**			
Chinese	8 (53.3)	3 (50.0)	11 (52.4)
Malay	1 (6.7)	2 (33.3)	3 (14.3)
Indian	1 (6.7)	0 (0.0)	1 (4.8)
Others	5 (33.3)	1 (16.7)	6 (28.6)
Comorbidities	3 (20.0)	2 (33.3)	5 (23.8)

**RACHS score**			
1	0 (0)	0 (0)	0 (0)
2	9 (60.0)	4 (66.7)	13 (61.9)
3	6 (40.0)	0 (0)	6 (28.6)
4	0 (0)	1 (16.7)	1 (4.8)
5	0 (0)	0 (0)	0 (0)
6	0 (0)	1 (16.7)	1 (4.8)

**STAT category**			
1	0 (0.0)	0 (0.0)	0 (0.0)
2	12 (80.0)	3 (50.0)	15 (71.4)
3	2 (13.3)	1 (16.7)	3 (14.3)
4	1 (6.7)	1 (16.7)	2 (9.5)
5	0 (0.0)	1 (16.7)	1 (4.8)
PIM 3	0.1 (0.1, 0.2)	0.2 (0.1, 0.7)	0.1 (0.1, 0.2)
PELOD 2	7.0 (5.0, 11.0)	5.5 (2.0, 9.0)	7.0 (5.0, 10.0)
Cardiopulmonary bypass time, min	193.0 (134.0, 269.0)	121.0 (82.0, 243.0)	187.5 (113.0, 263.5)
Aortic cross-clamp time, min	110.0 (65.0, 171.0)	33.0 (27.0, 34.0)	80.0 (37.0, 133.0)
Duration of surgery, min	335.0 (281.0, 428.0)	360.5 (263.0, 497.0)	354.0 (281.0, 428.0)
Intraoperative adrenaline	14 (93.3)	6 (100.0)	20 (95.2)
Intraoperative milrinone	14 (93.3)	4 (66.7)	18 (85.7)
Acute kidney injury	12 (80.0)	5 (83.3)	17 (81.0)
PICU mortality	1 (6.7)	1 (16.7)	2 (9.5)
ECMO	1 (6.7)	2 (33.3)	3 (14.3)
Length of PICU stay, days	2.0 (1.0, 6.0)	2.0 (1.0, 7.0)	2.0 (1.0, 6.0)
Length of hospital stay, days	10.0 (8.0, 21.0)	12.0 (5.0, 100.0)	10.0 (8.0, 21.0)

*Categorical and continuous variables presented as counts (percentages) and median (interquartile range), respectively*.

*PELOD, pediatric logistic organ dysfunction; PIM, Pediatric Index of Mortality; PICU, pediatric intensive care unit; RACHS, risk adjustment for congenital heart surgery, STAT, Society of Thoracic Surgeons-European Association for Cardio-Thoracic Surgery; ECMO, extracorporeal membrane oxygenation*.

**Table 2:** Summary of cardiac diagnosis and surgical procedures (*N* = 21).

Patient	Cardiac lesions	Surgical procedures	Corrective	Survived
1	Pulmonary atresia with ventricular septal defect, major aortopulmonary collaterals, left lower pulmonary artery stenosis	Repair of pulmonary artery stenosis (left main pulmonary artery to left lower pulmonary artery graft)	No	Yes
2	Hypoplastic right ventricle, large atrial and ventricular septal defect, transposition of the great arteries	Fontan procedure	Yes	Yes
3	Tetralogy of Fallot	Total repair	Yes	Yes
4	Pulmonary atresia with ventricular septal defect	Right ventricle to pulmonary artery conduitVentricular septal defect repair	Yes	Yes
5	Tetralogy of Fallot	Total repair	Yes	Yes
7	Tetralogy of Fallot	Total repair	Yes	Yes
8	Dextrocardia, hypoplastic right ventricle, transposition of the great arteries, severe pulmonary stenosis, moderate atrial septal defect	Fontan procedure	Yes	Yes
9	Tetralogy of Fallot	Total repair	Yes	Yes
10	Tetralogy of Fallot	Total repair	Yes	Yes
11	Pulmonary atresia intact ventricular septum, patent ductus arteriosus	Right ventricular outflow tract augmentationLigation of patent ductus arteriosus	Yes	Yes
12	Complete atrioventricular septal defect, hypoplastic aorta, coarctation of the aorta	Coarctation repair within 30 days	No	Yes
13	Pulmonary atresia with intact ventricular septum, moderate mitral regurgitation	Fontan procedureMitral annuloplasty	Yes	Yes
14	Congenitally corrected transposition of the great arteries, large ventricular septal defect, severe pulmonary stenosis	Right ventricle to pulmonary artery conduitVentricular septal defect repair	Yes	Yes
15	Tetralogy of Fallot	Total repair	Yes	Yes
16	Complete atrioventricular septal defect with moderate to severe atrioventricular valve regurgitation, total anomalous pulmonary venous drainage	Repair of complete atrioventricular canalRepair of total anomalous pulmonary venous drainage	Yes	No
17	Tricuspid valve atresia, hypoplastic right ventricle, ventricular septal defect	Bidirectional Glenn	No	Yes
18	Atrioventricular septal defect, hypoplastic right ventricle, pulmonary atresia, major aortopulmonary collaterals	Unifocalization for pulmonary atresia (right lower and middle major aortopulmonary collateral artery unifocalization)Systemic to pulmonary artery shunt (modified Blalock Taussig shunt)	No	No
19	Tetralogy of Fallot	Total repair	Yes	Yes
20	Hypoplastic right ventricle, large ventricular septal defect, pulmonary stenosis, transposition of the great arteries	Bidirectional Glenn	No	Yes
21	Hypoplastic left heart syndrome, partial anomalous pulmonary venous drainage	Stage 1 repair of hypoplastic left heart syndrome (Norwood operation)	No	No
22	Double outlet right ventricle–Tetralogy of Fallot type	Repair of double outlet right ventricle	Yes	Yes

All patients remained on mechanical ventilation on arrival to the PICU. Five [5/21 (23.8%)] were extubated within several hours of admission and 9/21 (42.9%) on postoperative day 1. The median duration of mechanical ventilation was 21.0 (13.0, 92.0) hours. Twenty [20/21 (95.3%)] patients required inotropic support on PICU admission. The most common inotrope used was milrinone [18/20 (90.0%)], followed by adrenaline [17/20 (85.0%)] (Table 3). Median lactate levels within the first 24 h was 2.7 (2.3, 5.0) mmol/L. Significant hemodynamic events occurred in 17/21 (80.9%) patients with the most common being hypotension [14/21 (66.7%)] (Table 4). Fluid bolus was required more frequently in the corrective surgery group in the first day of PICU compared to the second day [12/15 (80.0%) vs. 4/15 (33.3%); *p* = 0.022]. Acute kidney injury occurred in 17/21 (81.0%) patients. However, only one (5.6%) patient required renal replacement therapy. 3/21 patients required veno-arterial extracorporeal membrane oxygenation postoperatively, of whom 1/3 (33.3%) survived. Overall PICU mortality was 2/21 (9.5%). The causes of death in these two patients were necrotizing enterocolitis and hypoplastic left heart syndrome.

**Table 3:** Summary of intensive care unit (ICU) variables (*N* = 21).

ICU variables	Corrective surgery (*N* = 15)			Non-corrective surgery (*N* = 6)		
	Day 1 (*N* = 15)	Day 2 (*N* = 12)	*p*-value	Day 1 (*N* = 6)	Day 2 (*N* = 3)	*p*-value
Toe-core gap, °C	6.2 (4.0, 8.2)	6.7 (4.3, 7.8)	0.470	2.8 (2.8, 3.0)	5.8 (3.7, 8.0)	1.000
Central venous pressure, mmHg	12.3 (11.2, 13.0)	14.0 (8.0, 15.3)	0.278	8.5 (6.0, 10.4)	11.5 (9.3, 19.9)	0.500
Lactate, mmol/L	2.7 (2.3, 5.0)	1.9 (1.4, 3.7)	0.193	2.4 (1.3, 5.5)	2.0 (1.1, 3.8)	0.250
Hemoglobin, g/dL	13.0 (12.2, 14.5)	11.5 (10.7, 13.6)	0.148	11.6 (10.7, 12.6)	12.9 (10.1, 14.0)	1.000
Urine output, ml/kg/h	0.8 (0.6, 1.5)	1.1 (0.9, 2.2)	0.077	1.8 (1.1, 2.0)	1.2 (0.3, 4.1)	1.000
Cumulative balance, ml/kg	0.5 (−9.1, 10.9)	0.6 (−4.3, 19.1)	0.791	4.3 (−0.1, 115.7)^a^	93.7 (4.8, 130.5)^a^	1.000
Postoperative inotrope						
Dopamine	0 (0.0)	0 (0.0)	–	1 (16.7)	1 (33.3)	1.000
Adrenaline	14 (93.3)	6 (50.0)	**0.024**	3 (50.0)	2 (66.7)	1.000
Noradrenaline	2 (13.3)	3 (5.0)	0.628	1 (16.7)	2 (66.7)	0.226
Milrinone	15 (100.0)	10 (83.3)	0.188	3 (50.0)	2 (66.7)	1.000
Phentolamine	0 (0.0)	0 (0.0)	–	1 (16.7)	1 (33.3)	1.000

**Ventilation variables**						
PIP, cm H_2_O	17.2 (13.2, 19.3)	20.6 (19.0, 21.3)	0.688	18.0 (16.9, 20.3)	20.0 (18.0, 22.0)	1.000
PEEP, cm H_2_O	5.0 (5.0, 6.0)	6.3 (5.0, 7.0)	1.000	6.5 (5.0, 8.5)	8.0 (5.0, 10.0)	1.000
MAP, cm H_2_O	8.5 (7.4, 9.8)	10.9 (10.3, 11.4)	0.438	8.9 (8.5, 10.8)	10.4 (8.8, 12.0)	0.500
TV, mL/kg	7.9 (6.8,10.2)	6.6 (6.3, 6.8)	0.156	6.0 (4.1, 7.5)	3.7 (2.2, 5.1)	1.000
FiO_2_, %	0.4 (0.3, 0.7)	0.4 (0.3, 0.5)	0.734	0.3 (0.2, 0.4)	0.2 (0.2, 0.3)	0.500
SpO_2_, %	97 (95, 98)	98 (89, 99)	0.266	80 (77, 92)	90 (70, 98)	0.250
Inhaled nitric oxide	1 (6.7)	1 (8.3)	1.000	0 (0.0)	0 (0.0)	–

**Blood gas variables**						
pH	7.4 (7.4, 7.4)	7.4 (7.4, 7.4)	0.966	7.4 (7.3, 7.4)	7.4 (7.3, 7.4)	0.500
PaO_2_, mmHg	95.0 (65.8, 104.7)	93.7 (52.9, 117.6)	0.898	48.6 (44.4, 50.8)	77.2 (48.0, 100.1)	0.750
PaCO_2_, mmHg	39.1 (34.8, 40.8)	37.5 (34.6, 39.6)	0.520	44.4 (42.2, 48.1)	41.8 (39.8, 46.2)	0.250
HCO_3_, mmol/L	22.8 (21.8, 23.4)	22.6 (21.5, 23.3)	0.465	24.0 (22.0, 25.5)	23.8 (20.0, 27.5)	1.000
Base deficit	−2.3 (−3.6, −1.6)	−2.9 (−4.1, −2.0)	0.365	−0.1 (−2.8, 0.9)	−1.3 (−5.0, 4.3)	1.000

*Categorical and continuous variables presented as counts (percentages) and median (interquartile range), respectively*.

*^a^High third quartile due to two patients (number 18 and 21) with high cumulative positive balance*.

*PIP, peak inspiratory pressure; PEEP, positive end expiratory pressure; MAP, mean airway pressure; TV, tidal volume; FiO_2_, fraction of inspired oxygen; SpO_2_, pulse oximetry; PaO_2_, partial pressure of arterial oxygen; PaCO_2_, partial pressure of carbon dioxide; HCO_3_, bicarbonate*.

*Bold value represents p < 0.05*.

**Table 4:** Significant postoperative hemodynamic events and interventions within first 2 postoperative days.

	Corrective surgery (*N* = 15)			Non-corrective surgery (*N* = 6)		
	Day 1 (*N* = 15)*n*(%) nE	Day 2 (*N* = 12)*n*(%) nE	*p*-value	Day 1 (*N* = 6)*n*(%) nE	Day 2 (*N* = 3)*n*(%) nE	*p*-value
**Hemodynamic events**						
Hypotension	11 (73.3) 62	5 (41.7) 53	0.130	3 (50.0) 47	2 (66.7) 33	1.000
Desaturation	1 (6.7) 1	1 (8.3) 1	1.000	2 (33.3) 10	1 (33.3) 21	1.000
Cannula dislodgement	1 (6.7) 17	0 (0.0) 0	1.000	1 (16.7) 1	1 (33.3) 1	1.000
Arrhythmia	7 (46.7) 54	4 (33.3) 41	0.696	1 (16.7) 17	0 (0.0) 0	1.000
Cardiac arrest	0 (0.0) 0	0 (0.0) 0	–	0 (0.0) 0	0 (0.0) 0	–
Hemorrhage	3 (20.0) 9	2 (16.7) 3	1.000	0 (0.0) 0	0 (0.0) 0	–
Oliguria	1 (6.7) 2	0 (0.0) 0	1.000	0 (0.0) 0	0 (0.0) 0	–

**Interventions**						
Fluid bolus	12 (80.0) 51	4 (33.3) 21	**0.022**	2 (33.3) 17	1 (33.3) 5	1.000
Transfusion	5 (33.3) 19	2 (16.7) 3	0.408	3 (50.0) 31	2 (66.7) 13	1.000
Increase inotrope	3 (20.0) 6	3 (25.0) 4	1.000	1 (16.7) 2	2 (66.7) 7	0.226
Cardiac massage	0 (0.0) 0	0 (0.0) 0	–	0 (0.0) 0	0 (0.0) 0	–
Defirbillation/cardioversion	0 (0.0) 0	0 (0.0) 0	–	0 (0.0) 0	0 (0.0) 0	–
ECMO	0 (0.0) 0	0 (0.0) 0	–	1 (16.7) 25	1 (33.3) 24	1.000
Amiodarone	1 (6.7) 1	1 (8.3) 11	1.000	0 (0.0) 0	1 (33.3) 1	0.333
Chest toilet	1 (6.7) 1	0 (0.0) 0	1.000	0 (0.0) 0	0 (0.0) 0	–
Pacing	3 (20.0) 28	2 (16.7) 25	1.000	1 (16.7) 8	0 (0.0) 0	1.000

*Categorical and continuous variables presented as counts (percentages) and median (interquartile range), respectively*.

*ECMO, extracorporeal membrane oxygenation; *n*, number of subjects (% = percent of subjects); nE, total number events*.

*Bold value represents p < 0.05*.

In the corrective surgery group, SpO_2_ increased immediately post-CPB compared to pre-CPB [99 (98, 100) vs. 86% (79, 90); *p* < 0.001] and remained in the normal range through to hospital discharge [97% (92, 98)] (Table 5). Post-CPB CrO_2_ did not change from pre-CPB [72.8 (58.8, 79.0) vs. 72.1% (63.0, 78.3); *p* = 0.761]. Postoperative day 2 CrO_2_ was also not different from pre-CPB [68.3 (55.0, 74.4) vs. 72.1% (63.0, 78.3); *p* = 0.520]. On hospital discharge, however, CrO_2_ was lower than baseline (pre-CPB) [60.5 (53.6, 62.9) vs. 72.1% (63.0, 78.3); *p* = 0.005]. Of note, the majority of our patients [11/21 (52.4%)] had their baseline (pre-CPB) taken a few minutes after intubation and ventilation.

**Table 5:** SpO_2_, cerebral, and somatic tissue oxygenation measurements throughout the perioperative period.

	Time period						*p*-value (compared to pre-CPB)				
	CPB			ICU		Discharge	CPB		ICU		Discharge
	Pre	During	Post	Day 1	Day 2		During	Post	Day 1	Day 2	
**Corrective surgery**											
SpO_2_ (%)	86 (79, 90)	99 (94, 100)	99 (98, 100)	97 (95, 98)	98 (89, 99)	97 (92, 98)	**0.004**	**<0.001**	**<0.001**	**<0.001**	**<0.001**
Cerebral (%)	72.1 (63.0, 78.3)	69.2 (61.2, 74.7)	72.8 (58.8, 79.0)	66.4 (61.2, 75.1)	68.3 (55.0, 74.4)	60.5 (53.6, 62.9)	0.761	0.761	0.135	0.520	**0.005**
Somatic (%)	72.7 (65.6, 77.3)	86.3 (83.6, 88.6)	87.3 (77.2, 89.5)	72.5 (68.1, 79.3)	71.9 (63.4, 74.1)	66.9 (57.3, 76.9)	**<0.001**	**<0.001**	0.890	0.339	**0.048**

**Non-corrective surgery**											
SpO_2_ (%)	87 (79, 90)	95 (95, 95)	82 (82, 86)	80 (77, 92)	90 (70, 98)	80 (80, 81)	1.000	0.250	0.875	1.000	0.250
Cerebral (%)	59.4 (42.5, 65.8)	73.8 (66.0, 77.8)	58.8 (52.9, 67.7)	54.1 (46.4, 66.0)	64.4 (36.2, 66.6)	54.5 (52.9, 60.4)	0.125	0.875	0.875	1.000	0.250
Somatic (%)	64.6 (42.8, 74.7)	81.4 (74.1, 91.1)	79.2 (58.9, 84.1)	69.8 (60.4, 71.7)	65.3 (48.2, 66.1)	56.9 (50.3, 60.6)	0.125	0.125	0.625	0.500	0.250

*Continuous variables are presented as median (interquartile range)*.

*CPB, cardiopulmonary bypass; ICU, intensive care unit; SpO_2_, pulse oximetry*.

**Figure 2:**
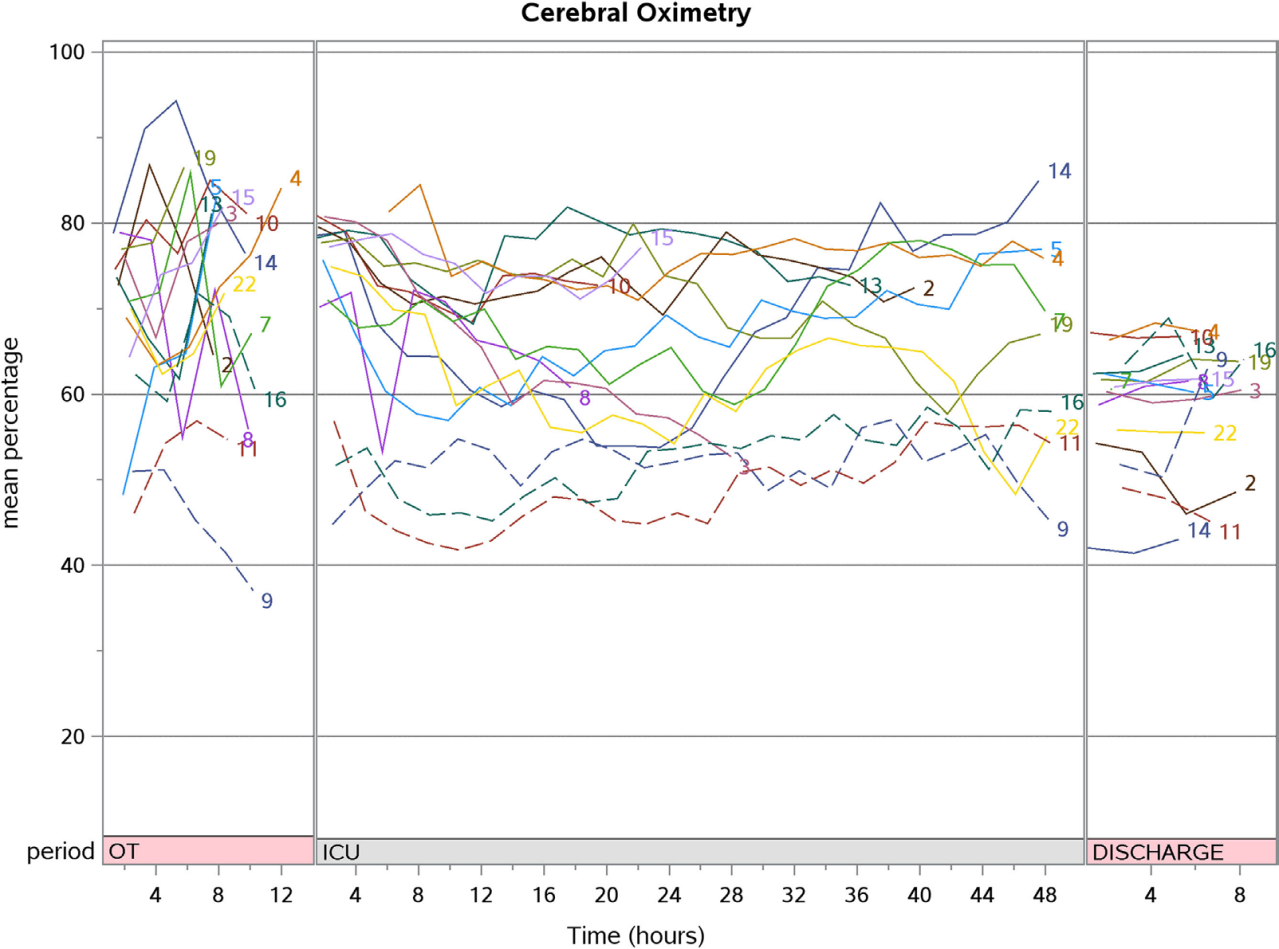
The aggregate cerebral oximetry trend for patients who underwent corrective surgery (*N* = 15).

**Figure 3:**
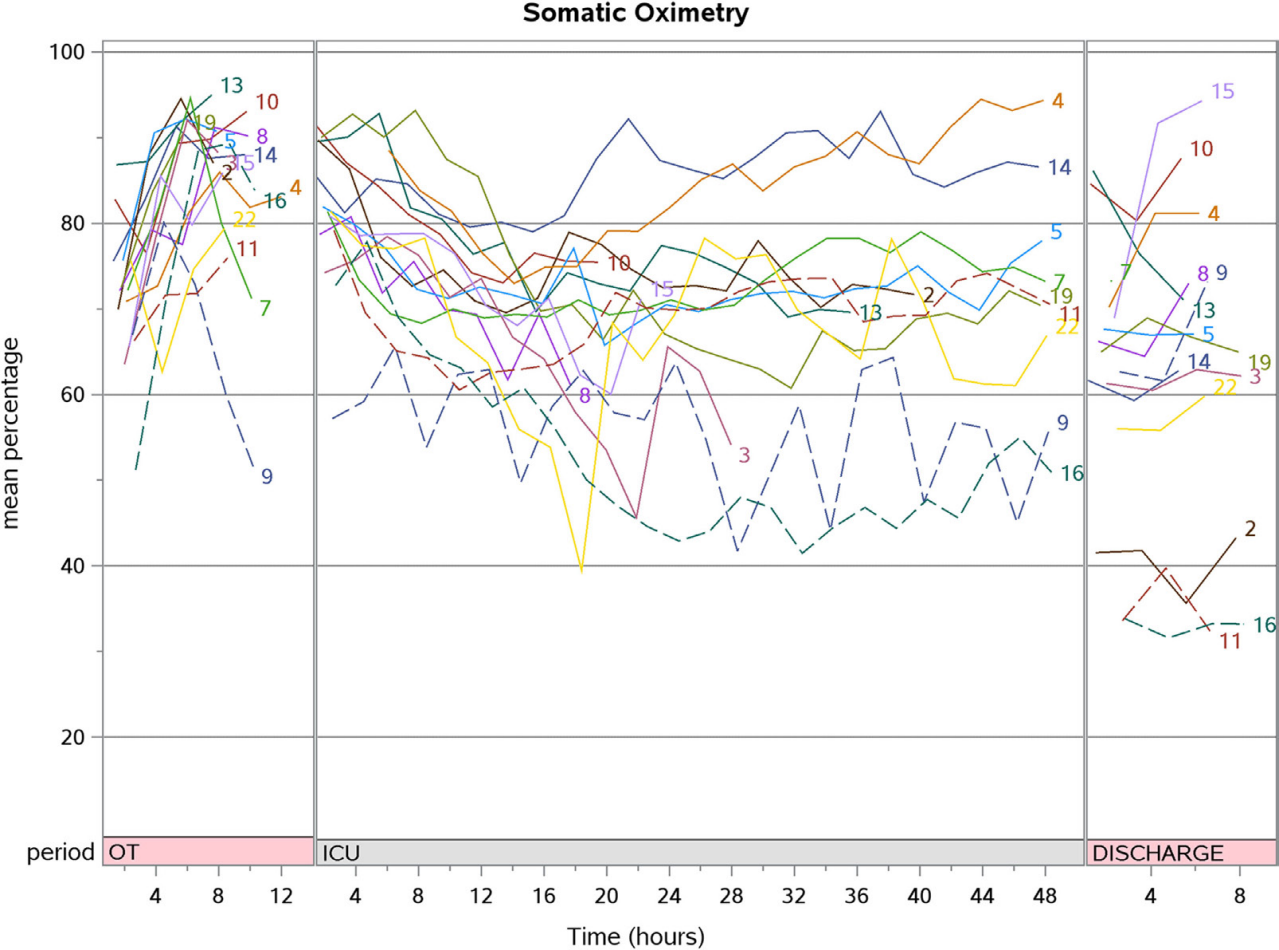
The aggregate somatic oximetry trend for patients who underwent corrective surgery (*N* = 15).

**Figure 4:**
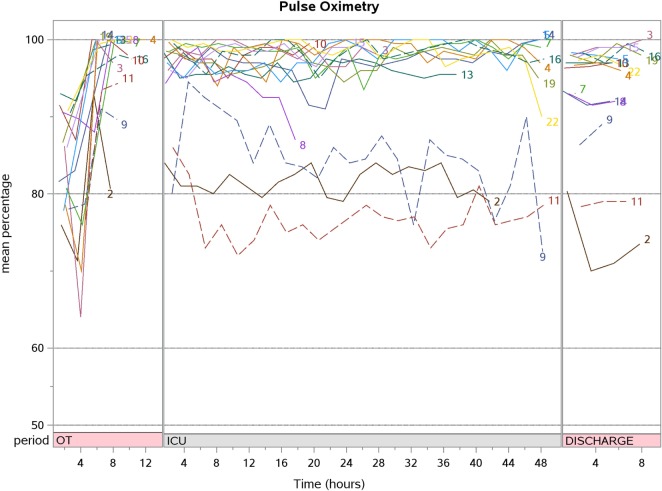
The aggregate pulse oximetry trend for patients who underwent corrective surgery (*N* = 15).

Post-CPB SrO_2_ increased compared to pre-CPB [87.3 (77.2, 89.5) vs. 72.7% (65.6, 77.3); *p* < 0.001] but progressively decreased during PICU stay to a value lower than baseline at hospital discharge [66.9 (57.3, 76.9) vs. 72.7% (65.6, 77.3); *p* = 0.048]. The aggregate SpO_2_, CrO_2_, and SrO_2_ trend for patients who underwent corrective surgery are shown in Figures 2–4. There were no significant changes in SpO_2_, CrO_2_, and SrO_2_ in the palliative surgery group from baseline, postoperatively and on discharge.

## Discussion

We prospectively studied a small cohort of 21 children with cyanotic CHD and characterized their CrO_2_ and SrO_2_ longitudinally over the perioperative period. Unlike SpO_2_ which normalized immediately after corrective surgery, CrO_2_ did not increase after surgical correction of cyanotic CHD even up to the time of hospital discharge. SrO_2_ showed a transient increment during and after CPB but decreased subsequently.

Arterial oxygen saturation measured by SpO_2_ increases after surgical correction of cyanotic CHD. CrO_2_, measuring the concentration of hemoglobin and oxy-hemoglobin in arterioles, capillaries and venules reflects cerebral tissue oxygenation which is taken as a surrogate of cerebral venous oxygenation (SvO_2_) (14, 15). In this observational study, the CrO_2_ remains the same. This seems to suggest that cerebral fractional oxygen extraction (cFOE) [cFOE = (SaO_2_ − SvO_2_)/SaO_2_] increased during the post-surgical repair period (16). These data are consistent with the literature which describes increased cerebral metabolic rate of oxygen (CRMO_2_) post-cardiac surgery (17). An observational study in term neonates with the primary objective of demonstrating CRMO_2_, arterial oxygen content, cerebral blood flow, and cFOE differences due to CPB duration and DHCA, reported increased CRMO_2_ [increase of 121% (59–207) from baseline; *p* = 0.17] and cFOE [increase of 145% (107, 222) from baseline; *p* = 0.005] 12 h after returning from the operating room (17). Taking these findings together, this may suggest that we should not target CrO_2_ above baseline after surgical correction. Of note, in the current study, only one patient underwent DHCA, which is associated with decreased CRMO and cFOE (18). Moreover, this patient died and did not contribute to hospital discharge CrO_2_ data. To the best of our knowledge, there are no studies describing cFOE trends in the postoperative period beyond the first postoperative day in children with CHD. As we were not able to measure cFOE directly, future studies describing these values in children undergoing cardiac surgery would be required to describe this physiological response in greater detail.

Baseline CrO_2_ and SrO_2_ in our cohort were 63.8 (57.2, 76.6) and 74.6% (68.9, 82.4), respectively. These values are relatively high compared to other studies involving children with cyanotic CHD. A large prospective study (*n* = 112) reported different baseline CrO_2_ with different diagnosis: tetralogy of Fallot (57 ± 12), pulmonary atresia (38 ± 6), single ventricle with aortopulmonary shunt (50 ± 7), bidirectional cavo-pulmonary connection (43 ± 6), and Fontan (70 ± 4). All patients except those in the Fontan group had CrO_2_ lower than the control group of healthy patients (68 ± 10) (10). In staged surgery for single-ventricle physiology, patients had a baseline CrO_2_ of 61% at pre-stage 1, 48% post-stage 1, CrO_2_ of 42% pre-stage 2, 53% post-stage 2, and CrO_2_ of 70% pre-Fontan which remained the same after completion of the Fontan procedure (11). We postulate the reason for the high baseline in our study is because the majority of our patients had their baseline (pre-CPB) taken a few minutes after intubation and ventilation. Patients in this cohort were mostly toddlers and young children who were uncooperative for placement of NIRS sensors prior to anesthesia. Hence, we postulate the high baseline tissue oximetry readings were because the patients were already sedated, intubated, and ventilated, and this may lead to increase in CrO_2_ and SrO_2_ readings (19, 20).

A retrospective study (*n* = 25) of infants undergoing elective cardiac surgery showed that CrO_2_ was always lower than that of other regions (including renal, arm, and thigh) (21). This reflects a higher FOE across the cerebral bed compared with the somatic bed (22). We observed a similar phenomenon in our cohort, and this supports the use of CrO_2_ as the main site for regional oximetry monitoring in the most vulnerable of patients. These patients while on CPB also showed a higher CrO_2_ and SrO_2_ compared to other perioperative time-points. This increase associated with CPB was observed regardless of anatomical diagnosis (10, 11, 23). In our study, we noted that there was an increase in both cerebral and SrO_2_ sites for on CPB. SrO_2_ was higher during CPB compared to pre-CPB [86.3 (83.6, 88.6) vs. 72.7% (65.6, 77.3); *p* < 0.001]. This effect normalized after coming off CPB. It is suggested that the marked increase in SrO_2_ site (above normal levels) is due to over-oxygenation or reduced oxygen consumption at these sites (21).

A trend of declining SrO_2_ is observed over the first 48 h as the patient recovers from surgery in the PICU. Although we were not able to elicit the exact reasons for this phenomenon in this study, this decreasing trend may be explained by factors that affect CrO_2_ and SrO_2_ such as increased oxygen demand after surgery as the patient is weaned from sedation and ventilation, low cardiac output states, hypovolemia, microcirculatory disturbance, increased vascular tone, and changes in the toe-core temperature gap (5, 19, 20). However, the lack of decline in CrO_2_ values may be related to the preservation of cerebral autoregulation (24, 25).

There was a decrease in both CrO_2_ and SrO_2_ at hospital discharge compared to baseline in both the corrective and palliative surgery groups, but it did not reach statistical significance for the latter possibly due to the small sample size. The oximetry readings taken at hospital discharge spanned 4–6 h during overnight sleep when the patient was considered well and fit for home. It is likely the CrO_2_ and SrO_2_ at hospital discharge reflects the true baseline and the pre-CPB values reflect a hyperoxygenated state as it was taken with the patient on mechanical ventilation.

Although this is a pilot and purely descriptive study, to the best of our knowledge, this is the first study to longitudinally examine NIRS perioperatively up to hospital discharge in children with cyanotic CHD undergoing surgical correction. We demonstrated that CrO_2_ and SrO_2_ did not show a sustained increase even up until the time of hospital discharge in patients who had undergone complete repair. Hence, our findings potentially imply that during the postoperative period, oximetry values may not be necessarily higher than baseline even when SpO_2_ completely normalizes. Even though our sample size was small, we managed to achieve our aim of describing the postoperative CrO_2_ and SrO_2_ values beyond 24 h after surgery. However, due to the small sample size we were not able to meaningfully stratify CrO_2_ and SrO_2_ according to baseline characteristics (e.g., age, type of procedure). Another factor may have limited our analysis was that our patients were young and too fretful to have the NIRS sensors placed pre-induction of anesthesia. Most of these sensors were placed after sedation and successful intubation, leading to higher baseline oximetry than expected. Another limitation is the wide limits of agreement between one cerebral oximeter and another, making devices non-interchangeable and hence, the results of this study may not be generalized to all makes of the cerebral oximeter (26). Finally, we were not able to obtain NIRS value during longer follow-up period such as 3–6 months after surgery, as we focused our study on the immediate perioperative period.

In conclusion, this pilot study demonstrated that SpO_2_ normalizes immediately after surgery but CrO_2_ and SrO_2_ does not show a sustained increase after surgical correction of cyanotic CHD even up to the time of hospital discharge. Future larger studies are required to validate our findings so as to inform future guidelines and management algorithms using NIRS in this group of children.

## Ethics Statement

This study was carried out in accordance with the recommendations of the SingHealth Centralised Institutional Review Board (reference number: 2015/2161). The study protocol was approved by the SingHealth Centralised Institutional Review Board. The legal representative of all subjects gave written informed consent in accordance with the Declaration of Helsinki.

## Author Contributions

JW, CC, RM, OW, TT, MN, JA, TL, and JL contributed conception and design of the study; RM and JA performed the statistical analysis; JW and JL wrote the first draft of the manuscript; CC, OW, TT, and MN wrote sections of the manuscript. All authors contributed to manuscript revision, read, and approved the submitted version.

## Conflict of Interest Statement

This study was investigator initiated but funded by Medtronic including the provision of the Somanetics NIRs device, neonatal, pediatric sensors, and the software necessary to conduct this study. JL is the principal investigator for the grant awarded by Medtronic for the conduct of this study (Project ID: ISR-2014-10423). Medtronic staff was not involved in the study design, execution, and analysis of the results.

## Abbreviations

CaO_2_, arterial oxygen content; CBF, cerebral blood flow; cFOE, cerebral fractional oxygen extraction; CHD, congenital heart disease; CPB, cardiopulmonary bypass; CRMO_2_, cerebral metabolic rate of oxygen; CrO_2_, cerebral oximetry; DHCA, deep hypothermic circulatory arrest; NIRS, near-infrared spectroscopy; PaO_2_, partial pressure of arterial oxygen; PICU, pediatric intensive care unit; SpO_2_, pulse oximetry; SrO_2_, somatic oximetry; SvO_2_, cerebral venous oxygenation.
